# An Unstructured Supplementary Service Data System for Daily Tracking of Patient Samples and Diagnostic Results in a Diagnostic Network in Malawi: System Development and Field Trial

**DOI:** 10.2196/26582

**Published:** 2021-07-06

**Authors:** Daniel Killian, Emma Gibson, Mphatso Kachule, Kara Palamountain, Joseph Bitilinyu Bangoh, Sarang Deo, Jonas Oddur Jonasson

**Affiliations:** 1 Operations Research Center Massachusetts Institute of Technology Cambridge, MA United States; 2 Riders 4 Health Lilongwe Malawi; 3 Kellogg School of Management Northwestern University Evanston, IL United States; 4 Health Technical Services Malawi Ministry of Health Lilongwe Malawi; 5 Indian School of Business Hyderabad India; 6 MIT Sloan School of Management Massachusetts Institute of Technology Cambridge, MA United States

**Keywords:** diagnostic networks, mobile phone, sample transportation, sub-Saharan Africa, data collection

## Abstract

**Background:**

Diagnostics in many low- and middle-income countries are conducted through centralized laboratory networks. Samples are collected from patients at remote point-of-care health facilities, and diagnostic tests are performed at centralized laboratories. Sample transportation systems that deliver diagnostic samples and test results are crucial for timely diagnosis and treatment in such diagnostic networks. However, they often lack the timely and accurate data (eg, the quantity and location of samples prepared for collection) required for efficient operation.

**Objective:**

This study aims to demonstrate the feasibility, adoption, and accuracy of a distributed data collection system that leverages basic mobile phone technology to gather reports on the quantity and location of patient samples and test results prepared for delivery in the diagnostic network of Malawi.

**Methods:**

We designed a system that leverages unstructured supplementary service data (USSD) technology to enable health workers to submit daily reports describing the quantity of transportation-ready diagnostic samples and test results at specific health care facilities, free of charge with any mobile phone, and aggregate these data for sample transportation administrators. We then conducted a year-long field trial of this system in 51 health facilities serving 3 districts in Malawi. Between July 2019 and July 2020, the participants submitted daily reports containing the number of patient samples or test results designated for viral load, early infant diagnosis, and tuberculosis testing at each facility. We monitored daily participation and compared the submitted USSD reports with program data to assess system feasibility, adoption, and accuracy.

**Results:**

The participating facilities submitted 37,771 reports over the duration of the field trial. Daily facility participation increased from an average of 50% (26/51) in the first 2 weeks of the trial to approximately 80% (41/51) by the midpoint of the trial and remained at or above 80% (41/51) until the conclusion of the trial. On average, more than 80% of the reports submitted by a facility for a specific type of sample matched the actual number of patient samples collected from that facility by a courier.

**Conclusions:**

Our findings suggest that a USSD-based system is a feasible, adoptable, and accurate solution to the challenges of untimely, inaccurate, or incomplete data in diagnostic networks. Certain design characteristics of our system, such as the use of USSD, and implementation characteristics, such as the supportive role of the field team, were necessary to ensure high participation and accuracy rates without any explicit financial incentives.

## Introduction

### Background

Most populations in sub-Saharan Africa rely on rural health clinics that have poor infrastructure as their primary point of entry to a broader health care system. These health clinics are often not equipped to conduct diagnostic testing, but they refer patient samples to a relatively small number of centralized laboratories capable of diagnostic analysis [1]. The effectiveness of programs targeting active diseases in the region, such as HIV/AIDS, tuberculosis (TB), and malaria, is therefore closely related to the performance of sample transportation (ST) systems put in place to transport patient samples and test results across the difficult terrain separating health facilities and molecular laboratories (MLs) [2].

ST systems in many sub-Saharan African countries operate without accurate information regarding the quantity and location of patient samples and test results requiring transportation [3]. Consequently, these ST systems operate in *push* mode, where couriers visit facilities on fixed weekly or biweekly schedules [3,4]. A common result of this operating mode is *empty trips*: courier visits to facilities where nothing was delivered to, or transported from, the facility. For example, an analysis of archival 2017-2018 courier data in Malawi revealed that 30.08% (24,256/80,648) of the courier visits to clinics were empty trips. This not only results in inefficient use of limited resources but also contributes to delays in receiving critical test results [5], which in turn leads to poor health-seeking behavior among the population [6] and can contribute to increased mortality rates [7].

An alternative to these ST push systems would be a *pull* system in which couriers only visit facilities when patient samples or test results are ready for transport to, or from, that facility. Such a pull system would limit empty trips but would require a reliable system to track the number of patient samples and test results requiring transportation across the diagnostic network. We hypothesize that these logistics data—specifically, the location and quantity of patient samples or test results ready for transport—can be collected with a low-cost information-sharing system and can be used to create an ST system that is responsive to real-time needs.

In this study, we investigated the feasibility, adoption, and accuracy of a system that leverages a communication protocol that is standard on all mobile phones, known as unstructured supplementary service data (USSD) technology [8], to gather more timely and accurate information. To conduct this investigation, we designed and developed a USSD collection system (hereafter, *the USSD system*) to enable health care facilities in a diagnostic network to report daily sample volume data. We conducted a year-long field trial of the USSD system in Malawi from July 2019 to July 2020 to determine whether our system would enable the timely collection of information.

### Intervention Development and Design

The 2 critical design questions we faced when developing our system were as follows:

1. What technology would health workers use to submit sample volume reports?
2. How would these reports be structured?

Given the positive findings regarding the feasibility of mobile health (mHealth) initiatives in low- and middle-income countries [9] and the rapid growth in mobile network coverage in sub-Saharan Africa over the past decade [10,11], we designed our system so that health workers could submit reports with a mobile phone. As less than half of the mobile phones in the region are smartphones [11], we based our sample volume data collection system on USSD technology—a mobile communication protocol accessible to both smartphones and less technologically advanced feature phones.

In a USSD system, users are provided a unique numeric access code that they can dial to access a structured menu of options. With an appropriate series of key presses using the mobile phone’s keypad, users can navigate through the options menu and submit information, similar to the method that an SMS text messaging system uses to convey information through text. One advantage of USSD over SMS text messaging, especially in the context of data collection, is that the USSD’s built-in menu system allows for structured responses, leading to built-in data validation [8]. This also reduces the amount of effort required to leverage the data systematically, which is crucial for managing the growing volume of health care data received through mobile phones [12].

**Figure 1:**
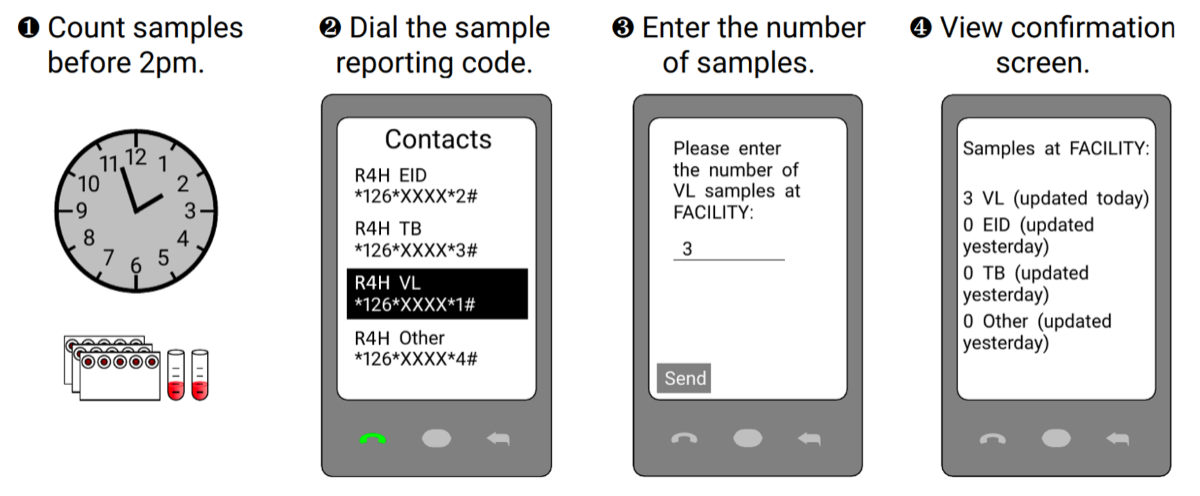
Unstructured supplementary service data system reporting instructions. (1) A health worker counts the number of samples, by sample type, that are prepared for transport. (2) The health worker dials a USSD code specifically assigned for the health worker's clinic and the type of sample (EID, VL, TB, and other), which has been saved on the health worker's phone and is displayed on a poster in the health facility. (3) The health worker enters the number of samples currently awaiting transportation at the facility. (4) The health worker views a confirmation screen, which summarizes how many samples of each type have been reported for the facility. EID: early infant diagnosis; R4H: Riders for Health; TB: tuberculosis; USSD: unstructured supplementary service data; VL: viral load.

In our USSD system, a specific USSD reporting code is assigned to every health facility operating within a diagnostic network, and each diagnostic test offered in the network is assigned a unique numeric designator. To report the number of patient samples for a specific test, a designated health worker at a designated facility can use any mobile phone to dial that facility’s USSD reporting code and the numeric designator for the diagnostic test to connect to the USSD system. Once connected, they can report the number of patient samples through a text-based interface. Upon entering all the required information, the user receives a confirmation text message containing a summary of the submitted report. Refer to Figure 1 for further details.

## Methods

### Study Setting

As of 2018, an estimated 9.75% (790,000/8,100,000) of the adults (aged 15-49 years) in Malawi were living with HIV [13], and the country’s TB incidence rate was 181 per 100,000 people [14]. The Malawi Ministry of Health (MOH) operates a diagnostic network of approximately 700 widely distributed community health clinics, 27 centrally located district health offices, and 10 regionally aligned MLs. The structure of the Malawi diagnostic network is representative of the diagnostic networks of many other countries in the region [2].

Since 2016, the Malawi branch of the nonprofit organization Riders for Health International (R4H) has managed the transportation of viral load (VL) samples, early infant diagnosis (EID) tests, and TB samples from health clinics and district health offices to the MLs. R4H maintains a team of more than 70 motorcycle couriers who visit health facilities and laboratories according to fixed weekly schedules.

In collaboration with MOH and R4H, we identified 3 districts in Malawi to test the USSD system: Salima in the central region, Rumphi in the northern region, and Phalombe in the southern region. The diagnostic networks in these rural and semirural districts each contained between 15 and 18 facilities and relied upon one ML to conduct the diagnostic testing for that diagnostic network, making these districts a representative sample of R4H’s typical ST operations in rural and semirural areas.

This field trial was approved by Malawi’s National Health Sciences Research Council. An evaluation by the Massachusetts Institute of Technology Committee on the use of humans as experimental subjects determined that the trial did not constitute human subjects research, as defined in Federal Regulations 45CFR46.

### System Implementation

In early 2019, we contracted with a local vendor in Malawi to develop the user interface and information technology infrastructure. The vendor also managed the daily operation of the system, which included storing incoming data, sending reminder messages when appropriate, and contracting with the cellular network providers to enable free provision of the USSD service to health workers.

In May 2019, we asked the facility in-charge from each of the participating health facilities to nominate 1 or 2 staff members with personal mobile phones to enter the data. In June 2019, we conducted three 2-hour training sessions (1 per study district) to train 150 health workers to use the USSD system. During the training sessions, we introduced the USSD system to the participants, taught the study participants how to access the system and submit reports using their personal mobile device, and provided reference posters and flyers that reminded the participants how to access the system for them to display at their facilities. A field team, consisting of a local field manager and 3 local research assistants, monitored the implementation and addressed technical and logistical challenges through regular communication with health workers, district laboratory technicians, and R4H couriers through phone calls and text messages.

The USSD system was officially launched in the study districts in July 2019. Health workers were asked to report the number of patient samples waiting to be transported at the end of each day. Facilities were expected to submit a report every day, even if they had not collected any samples or prepared any new samples for transportation. Health workers and field team members were sent a series of automated daily reminder messages to increase participation (Textbox 1).

**The daily schedule of reminder notifications.**

**Daily Reminder Notifications: Notification Delivery Time and Content**

- 8 AM: Health workers at each facility are sent a message notifying them whether or not a courier will visit their facility later that day as well as a reminder to report sample volumes.
- Noon: Health workers at each facility are sent a second reminder to report sample volumes.
- 1:30 PM: Members of the field team are sent a summary of the facilities for which a report has or has not been submitted.
- 2:15 PM: Health workers at facilities missing all or part of a complete daily report are reminded to report sample volumes.
- 3 PM: Members of the field team are sent an updated summary of the facilities for which a report has or has not been submitted as well as a comparison of each facility’s current report with its previous report (because an unusual increase or decrease from the previous report may indicate that the facility is reporting incorrectly).
- 4:15 PM: Members of the field team are sent a notification informing them whether couriers have submitted reports to the courier database. Reports submitted to the courier database are summarized by facility, compared with that facility’s most recent unstructured supplementary service data (USSD) report, and sent to members of the field team to assess facility reporting accuracy.
- 7 PM: Members of the field team are sent an updated list of the couriers who have or have not submitted reports to the courier database. A summary of reports submitted to the courier database by the facility are recompiled to capture any updates, recompared with that facility’s most recent USSD report, and sent to members of the field team to assess facility reporting accuracy.

On the basis of the notifications received from the health facilities, the field team sought out any unusual participation patterns such as intermittent, erratic, or extended periods of no participation. Upon detection of unusual participation patterns, the field team was authorized to directly address these patterns with the participant. In situations where an ordinarily reliable participant simply forgot to report or health workers in the same facility failed to properly delegate reporting responsibilities, the field team could directly contact the designated staff member at the facility. The field team could then remind the designated staff member to submit their daily report or to delegate reporting responsibilities to a different health worker when the primary contact was not at the facility. If the field team identified intermittent network coverage as the cause of a missed report, the field team could delegate reporting responsibility to someone with a network connection. In addition, the field team could also ask the courier to hand-deliver a message to the responsible health worker, to identify someone else at the facility to accept reporting responsibilities, or to request escalation to a higher authority at the nonreporting facility in email notifications regarding their facility’s participation.

The field team also monitored the accuracy of reports and intervened directly with the participants if they observed a pattern of low-accuracy reports. As in the case of poor participation, the field team could use a combination of phone calls, text messages, and hand-delivered messages to identify the root causes of data inaccuracies and address them. The preferred approach for improving reporting accuracy was to provide additional instructions to the noncompliant participant. If a training update failed to address the situation, more drastic measures (eg, requesting transfer of reporting to another staff member) could be adopted.

### Evaluation Framework

As part of the USSD system implementation plan, we elected to evaluate the feasibility, adoption, and accuracy of the USSD system using relevant descriptive statistics. Feasibility and adoption are common evaluation domains in the intervention assessment literature [15,16]. Accuracy, although not a common evaluation domain regarding health interventions, is relevant in the context of mobile device–based data collection systems [17]. Textbox 2 lists the guiding questions and associated metrics for assessing the system’s performance within the 3 domains. The feasibility of the USSD system depended on whether each facility had access to the technology required to engage with it. Therefore, we identified the number of facilities employing someone with a mobile device who was willing to participate in the study and the number of facilities in the field trial districts receiving service from a wireless network provider.

To assess the adoption of the USSD system, we monitored specific participation-related metrics: percentage of facilities reporting by day, individual facility participation over the course of the field trial, and the longest period that each facility went without participating.

We determined the accuracy of the USSD system by comparing the submitted USSD reports with program data. A data report was deemed accurate if the reported number of patient samples of a given type ready for delivery and the actual number of patient samples ready for delivery, as determined by the couriers, were identical.

**Evaluation framework.**

**Domains, Guiding Questions, and Metrics of the Evaluation Framework**

**Feasibility**

- Do facilities have access to mobile devices?
  The fraction of facilities for which a personal mobile phone was registered.
- Do facilities receive a mobile network signal?
  The fraction of facilities where insufficient network connection never prevented that facility from submitting a report.
  The fraction of facilities where staff members at that facility submitted a daily report for at least 7 consecutive days.

**Adoption**

- Are facilities participating?
  The fraction of facilities that reported or failed to report each day by sample type.
  The fraction of total reporting days over the trial period when each facility reported or failed to report.
  The largest number of consecutive days that a facility failed to report.
- Which operational factors influenced facility participation?
  The fraction of facilities where an insufficient understanding of the unstructured supplementary service data (USSD) system on the part of health workers prevented USSD participation.
  The fraction of facilities where hardware limitations prevented USSD participation.
  The fraction of facilities where health worker workload prevented USSD participation.
  The fraction of facilities where health worker absences prevented USSD participation.
  The fraction of facilities where health worker forgetfulness prevented USSD participation.

**Accuracy**

- How accurate are the data reported by participating facilities?
  The average and variance of the difference between reported and actual sample volumes.

### Data

We used data from 4 distinct sources to calculate the metrics listed in Textbox 2: the USSD system database, the courier database, a survey administered to members of the field team, and the attendance roster from the USSD system training sessions.

Every data report submitted through the USSD system during the field trial was archived in the USSD system database. This database included the facility name, date and time, the user’s mobile number, sample type, and number of patient samples reported by the user for every data report.

The courier database contained sample-specific information submitted by the couriers upon completion of the couriers’ daily routes to a data collection system operated by R4H. The sample data captured in the courier database consisted of the sample’s identification code, the name of the facility the sample originated from, the date of sample collection, and the date of sample pick-up from the originating facility.

Upon completion of the study, we administered a survey to the research assistants to assess the barriers to system participation. For each of the 51 facilities included in the field trial, the local research assistants were asked the questions presented in Textbox 3.

We compiled a master attendance roster by combining the individual attendance rosters recorded at each of the 3 USSD system training sessions. These rosters included the name of each training participant, the facility the participant represented, the participant’s staff position, and the participant’s contact information.

**Questions asked in the survey to research assistants.**

**Survey Questions Answered by Each Research Assistant**

- For each facility in your district, estimate the number of times poor network reception prevented the facility from reporting.
- Rate the effectiveness of the following techniques on participation by facilities in your district:
  SMS text messages
  Individual messages via a popular internet messaging platform
  Phone calls
  Asking a courier to deliver a message
  In-person facility visits
  Group messages via a popular internet messaging platform

### Analysis

To calculate the percentage of facilities for which a personal mobile phone was registered, we reviewed the master training attendance roster. Attendance at a training event by an employee from a given facility indicated that the employee owned a mobile phone and was willing to use their device to submit data reports to the USSD system. To calculate the percentage of facilities with a sufficient network connection, we summarized the survey responses regarding the frequency with which poor network connectivity prevented each facility from participating.

To determine the number of facilities for which a USSD report was submitted for at least 7 consecutive days, we analyzed the facility name and date of every report submitted to the USSD system database. Aggregation and summarization of data in the USSD system database also allowed us to measure all 3 metrics associated with the first guiding question in the adoption domain (Textbox 2).

We calculated the accuracy of the reported data by comparing the reported data in the USSD system database with the courier reports in the courier database, which captured the number of patient samples collected from the health care facilities.

All data analyses were conducted using the R (v 4.0.0; R Foundation for Statistical Computing) language and RStudio Desktop (v 1.2.5042). Reported *P* values were calculated using 1-sided nonparametric Mann-Whitney tests (unless otherwise noted).

## Results

### Descriptive Statistics

Over the study period (July 2019 to July 2020), the participating facilities submitted 37,771 reports to the USSD system, accounting for 48,852 patient samples. Most of these patient samples (40,952/48,852, 83.83%) were VL samples, whereas 6.1% (2979/48,852) were EID samples, 5.85% (2859/48,852) were TB samples, and 4.21% (2056/48,852) were classified as *Other*. Of the samples reported, 43.71% (21,355/48,852) originated in Phalombe, 35.12% (17,155/48,852) originated in Salima, and 21.17% (10,342/48,852) originated in Rumphi. The table included in Multimedia Appendix 1 contains sample volume statistics by district.

### Feasibility

All participating facilities employed at least one individual willing to submit reports to the USSD system with a personal mobile device. The research assistants reported that an insufficient network connection never prevented 47% (24/51) of the participating facilities from submitting a report to the USSD system, caused occasional submission problems in 24% (12/51) of the facilities, and caused frequent problems in 29% (15/51) of the facilities. Our analysis of the USSD database data also revealed that each facility had at least one 7-day period during which the facility submitted a report every day.

### Adoption

**Figure 2:**
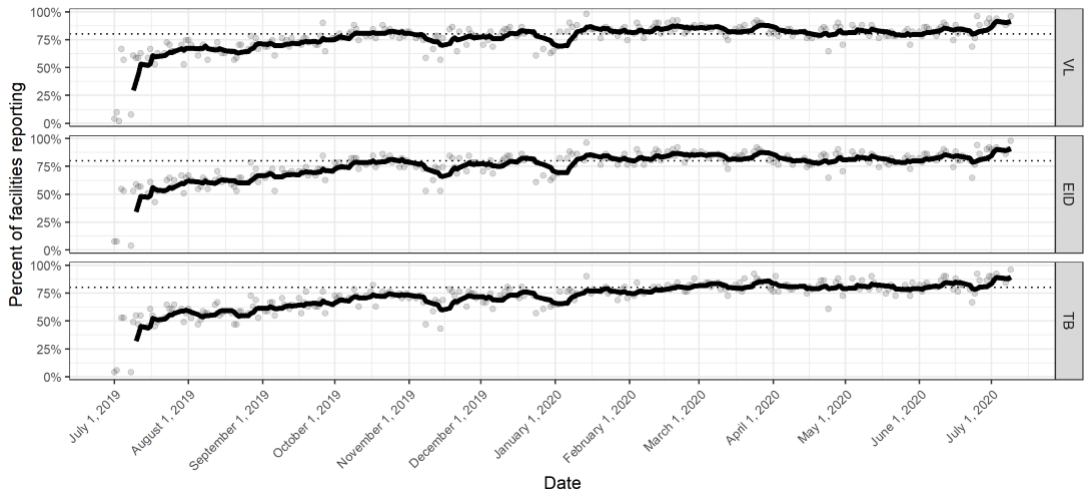
Facility participation by sample type, shown as daily participation percentages and as a 7-day moving average. EID: early infant diagnosis; TB: tuberculosis; VL: viral load.

Figure 2 illustrates the daily sample reporting rates and the 7-day moving average of daily sample reporting rates for the 3 patient sample types between July 2019 and July 2020. At the beginning of the study, only 10% (5/51)-20% (10/51) of the facilities participated each day. However, after 3 weeks, the daily participation rates increased and remained between 53% (27/51) and 98% (50/51) for VL samples, between 51% (26/51) and 98% (50/51) for EID samples, and between 43% (22/51) and 96% (49/51) for TB samples. Between August 2019 and January 2020, the 7-day moving average participation rate increased gradually for VL (from 63% to 87%), EID (from 60% to 85%), and TB (from 54% to 79%) samples, with notable but temporary declines during the second halves of November and December. For the final 5 months of the trial (February 2020 to July 2020), the average participation rate remained at or above 75% (38/51) for all 3 sample types.

**Figure 3:**
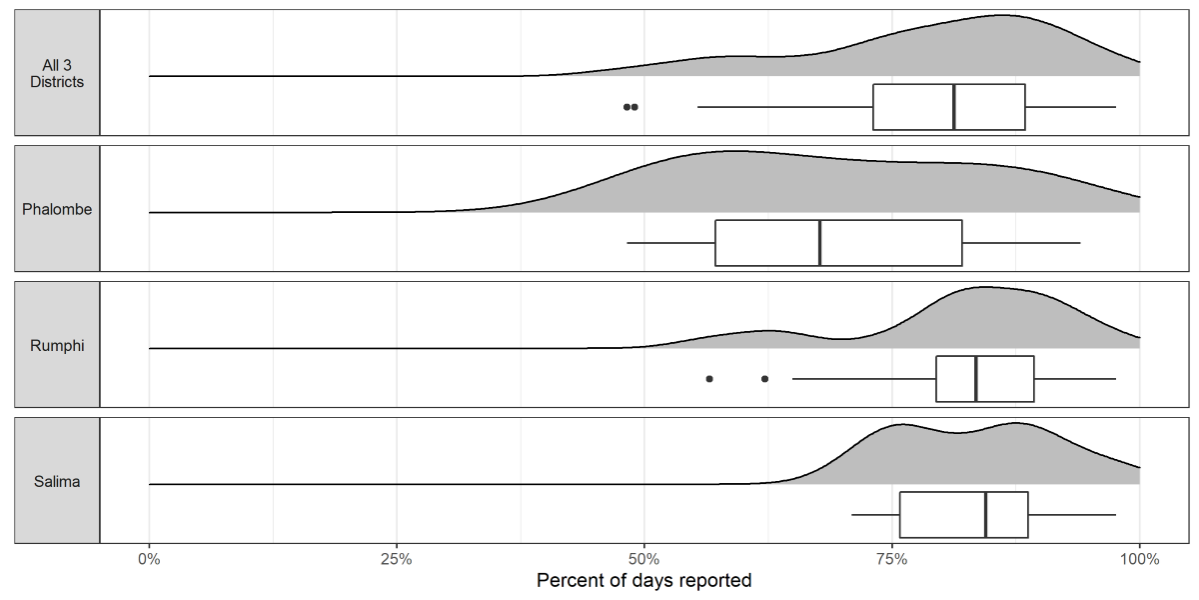
Distribution of facility reporting frequency.

The distribution of the facility participation rates across all districts and by individual districts, where the facility participation rate is calculated as the percentage of days out of the total possible reporting days for which a facility reported, is shown in Figure 3. On average, the facilities provided a report 78.9% (198/251 possible reporting days) of the time (σ=32.6 days). The median number of days a facility reported was 81.3% (204/251) days, with a range from 48.2% (121/251) days to 97.6% (245/251) days. The facilities in Phalombe reported less frequently than those in Salima (*P*=.003) and Rumphi (*P*=.01) on average.

Of the 51 health facilities, 45% (23/51) never went more than 1 business week (5 days) without submitting a report, and the longest any facility went without providing a report was 30 days. On average, the longest a facility went without submitting a report was 8.65 days (σ=6.33 days).

Table 1 shows the frequency of participation challenges faced by health facilities according to the 3 local research assistants. Each cell shows the number and percentage of facilities reported to experience a specific concern to the given extent. Recurring compliance issues were most often due to poor network reception, whereas the occasional noncompliance issue was most likely due to forgetfulness on the part of the health worker. Staff absence at the participating facilities also caused reporting issues at most (31/51, 61% facilities) of the participating facilities. These research assistant survey results also suggest that health workers had an adequate understanding of the system; poor staff training did not cause any reporting problems in more than 80% (41/51) of the facilities.

**Table 1:** Results regarding the causes of reporting issues at each of the 51 participating facilities (according to the 3 research assistants; n=51).

Reason for no report	Facilities, n (%)		
	No problems	Occasional problems	Frequent problems
Insufficient mobile network reception	24 (47)	12 (24)	15 (29)
Phone issues (eg, low battery or broken phone)	29 (57)	18 (35)	4 (8)
Staff are absent	20 (39)	21 (41)	10 (20)
Staff are too busy	26 (59)	15 (29)	6 (12)
Staff do not understand how to use the system	43 (84)	8 (16)	0 (0)
Staff forgot to report	4 (8)	39 (76)	8 (16)

### Accuracy

**Figure 4:**
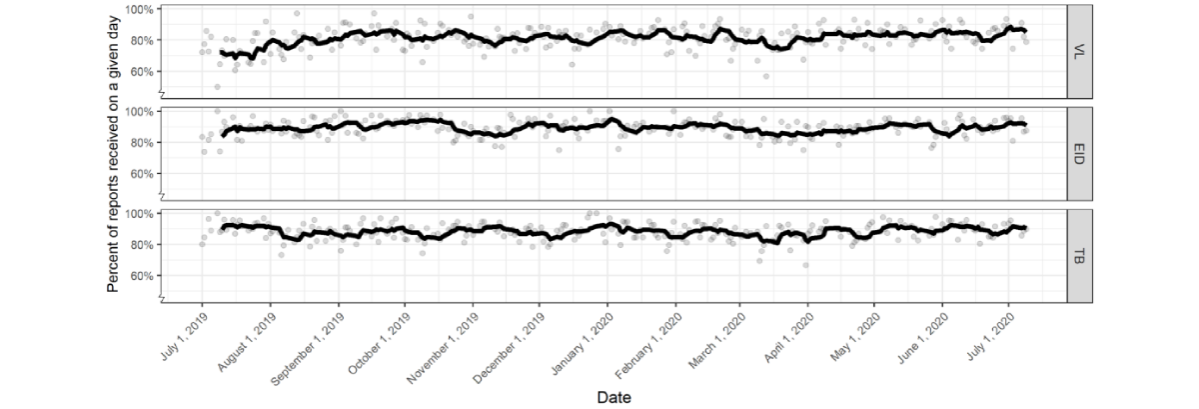
The number of accurate reports by sample type, shown as daily accuracy percentages and as a 7-day moving average. EID: early infant diagnosis; TB: tuberculosis; VL: viral load.

Figure 4 illustrates the daily percentage accuracy of reports for each sample type and its 7-day moving average. The daily accuracy of VL reports slowly improved over the first 2 months of the field trial and settled at approximately 80% for the remainder of the trial. Unlike the daily accuracy of VL reports, that of EID and TB reports did not change substantially throughout the trial, with the daily accuracy of VL reports exhibiting greater variance (σ=7.23) than both EID daily reporting accuracy (σ=5.25; *P*<.001 according to Levene test) and TB daily reporting accuracy (σ=5.38; *P*<.001 according to Levene test). On average, 81% of the daily VL reports, 89.2% of the daily EID reports, and 88.2% of the daily TB reports were accurate.

**Figure 5:**
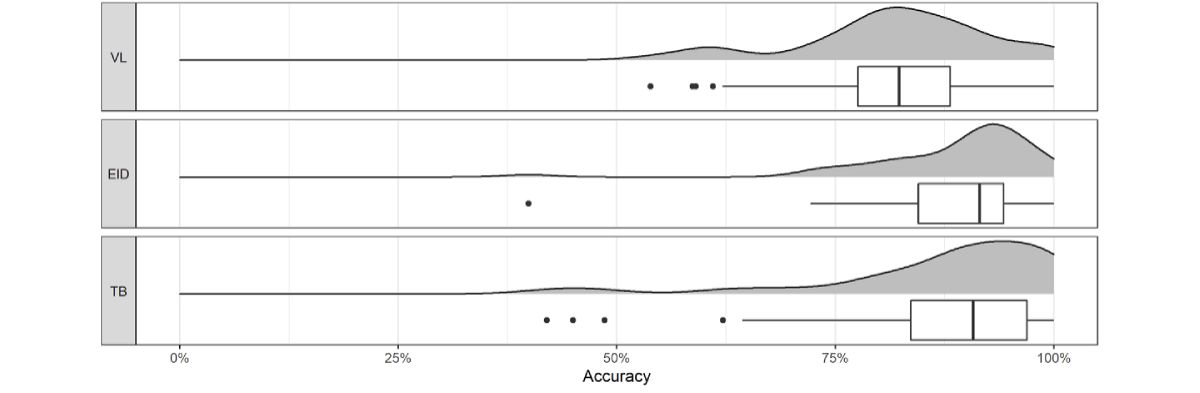
Distribution of facility reporting accuracy by sample type. EID: early infant diagnosis; TB: tuberculosis; VL: viral load.

The distribution of data accuracy by sample type across facilities is shown in Figure 5. The accuracy of EID reports exhibited the least variation (σ=0.01), followed by VL reports (σ=0.11) and TB reports (σ=0.14). The median facility reporting accuracy for VL was 82%, which was lower than that for EID (91%; *P*=.001 according to a paired Mann-Whitney test) and TB (91%; *P*=.001; paired Mann-Whitney test). For each sample type, more than half of the facilities submitted accurate reports on more than 80% of the days in the field trial.

## Discussion

### Principal Findings

#### General Findings

We designed a system whereby staff members in geographically dispersed health care facilities could report, through any mobile device, the number of patient samples prepared for delivery to a diagnostic test facility. Between July 2019 and July 2020, we conducted a field trial of the system in 3 districts in Malawi to assess the system’s feasibility, adoption, and accuracy. The results from our field trial suggest that the USSD system is a feasible, adoptable, and accurate tool for assembling accurate daily reports on the quantity and location of transportation-ready patient samples and diagnostic test results.

#### Feasibility

The feasibility of the USSD system is driven by the ease with which facilities can access it for report submission with a mobile network connection and a mobile device. Although mobile network coverage varies by country, our findings with respect to the number of facilities able to submit a report through the system illustrate the potential of using mHealth systems to link rural health facilities to central operations managers in sub-Saharan Africa, especially as mobile network coverage continues to improve across the region [11]. We also found at least one person at each facility with a mobile phone—a significant result because less than half of the population in the region owns a mobile phone [18]. Although prior work in the region regarding mHealth initiatives among the general population has, at times, found poor eligibility rates among potential participants driven by low mobile phone ownership [19,20], our findings suggest that mHealth efforts requiring ownership among health workers may be more feasible than those requiring ownership among the general population [21]. In addition, it is likely that the use of USSD in a region where smartphones constitute less than 40% of all mobile phones with lower-tier connections (ie, 1G/2G as against 4G/5G) also contributed to system feasibility [11]. The use of health workers’ personal phones avoided the additional cost of deploying new devices in the field and leveraged their familiarity with their devices to enhance system usability and transfer maintenance responsibilities to them [22].

#### Adoption

The adoption of the USSD system improved consistently over the course of the study and peaked near 90% toward the conclusion of the study, with the exception of small and temporary declines coinciding with personnel transitions (ie, staff reallocations and annual training sessions) and holiday seasons. These findings are comparable with results from similar mHealth studies conducted in the region [21,22], despite our study requiring more frequent reporting than other studies and doing so without monetary compensation for the participants. On the basis of the calculated descriptive statistics, we attribute the wide adoption of the USSD system to close collaboration with MOH representatives. This collaboration secured the support of senior government officials who encouraged participation by health workers at the facilities. In addition, this collaboration improved the chances of the system’s design complementing health workers’ existing responsibilities rather than adding to them, which is known to increase the likelihood of system adoption [19,21,23]. The efforts of the field team members in their role as real-time participation monitors and problem solvers likely also influenced the observed participation rate.

#### Accuracy

The existence of the courier database and our ability to access that data played a significant role in ensuring the high accuracy of the records (>80%). In contrast with prior mHealth initiatives [22,24], the courier database allowed us to assess the accuracy of every report submitted through our system with minimal delay and to provide feedback, via the field team, to correct improper reporting behavior daily. Providing timely and relevant feedback to a health worker regarding their reporting behavior likely contributed to the overall reporting accuracy achieved in the field trial [25].

### Strengths and Limitations

The scope of our study is limited to establishing the feasibility, adoption, and accuracy of a USSD system for collecting information on the quantity and location of patient samples and test results prepared for delivery in the diagnostic network. Therefore, the impact of making these data available on subsequent operational decisions or on patient care remains undetermined. This information is expected to be useful for avoiding unnecessary health facility visits, but there are many ways to incorporate the information provided by the USSD system into an operational courier routing system aimed at reducing empty trips and delays. The rigorous quantification of the effect of the USSD system on ST operations is beyond the scope of this study. However, a recent report by Gibson et al [26] indicated that this information, along with a sophisticated routing system, can reduce empty trips by at least 55%.

In addition, the results presented in Table 1 regarding the operational factors affecting facility participation are based on data collected indirectly through a survey administered to the 3 research assistants in the field. Ideally, these data should have been collected directly from each facility because this would have provided more granular information about the operational drivers for participation. However, collecting operational data on a daily basis was beyond the scope of this effort, and the constant rotation of health workers into and out of the facilities over the course of the study made it infeasible to conduct an end-of-study survey at each facility. We believe that surveying the local research assistants was the next best solution because they were in regular contact with multiple health workers from each facility and were aware of their experiences with the USSD system. Regardless of this limitation, the main objective of this study was to assess the feasibility, adoption, and accuracy of collecting information using the USSD system, all of which can be evaluated using primary data sources.

A notable strength of our study is that it demonstrates a novel use of mHealth technology to significantly improve information sharing in diagnostic networks, which have a similar structure in many low- and middle-income countries. Previous mHealth studies have investigated how mHealth technology can improve health care delivery through the wide dissemination of health-related information [9], providing patient-specific reminders and/or results to patients [27,28], connecting health care providers at different levels in the health care network [4,29], and monitoring medical supply stock levels [21,22,29], among other applications [19,30]. To the best of the authors’ knowledge, this study represents the first application of mHealth technology to track the location of samples and results in a large-scale diagnostic network. Additional strengths of this study include the fact that field implementation was sustained for an entire year, and that the structure of the system allowed us to determine the accuracy of every submitted report.

### Scalability

The USSD system is extremely scalable from a technological perspective because there is no requirement to purchase a specific mobile phone, mobile phone airtime, or any other system-specific technology. Expanding the system to operate with new facilities and/or new diagnostic tests simply requires assigning USSD identification codes and training new users, which itself is not very onerous because of the familiarity of mobile users in the region with USSD technology through other apps [8]. The scalability of the USSD system is further enhanced because it does not require the purchase of new hardware or software. The system operates with a mobile network signal that is increasingly available across most of the region and uses technology universally embedded in all mobile devices.

As explained earlier, the USSD system was designed to minimize impact on health workers’ workload, which should positively affect adoption in other health facilities without disrupting their routines [31,32]. Furthermore, health workers who currently use the USSD system can share their experiences in training sessions for new facilities, thereby further speeding up adoption. However, scalability may be adversely affected by continued reliance on field teams for data monitoring and supervision. As we work on developing scale-up plans in collaboration with R4H and MOH, we believe that incorporating these tasks into the roles of senior personnel within the ST systems, such as the regional ST coordinators who oversee ST operations within the districts, can help overcome this problem and facilitate scalability.

### Conclusions

Malawi’s diagnostic network, both in terms of the network’s structure and challenges, is representative of many diagnostic networks being operated in sub-Saharan Africa [2]. The descriptive results of our study suggest that a USSD-based system is a feasible, adoptable, and accurate solution to the challenges of untimely, inaccurate, or incomplete data present in these diagnostic networks. The scalability of the USSD system, along with the promising results of our study, suggests that system implementation at the national level in many sub-Saharan nations is feasible and worthwhile.

## Appendix

Multimedia Appendix 1 Summary statistics regarding the number of samples, by type, district, and facility, included in the unstructured supplementary service data system database from July 2019 to July 2020.

## Abbreviations

EID: early infant diagnosis
mHealth: mobile health
ML: molecular laboratory
MOH: Ministry of Health
R4H: Riders for Health International
ST: sample transportation
TB: tuberculosis
USSD: unstructured supplementary service data
VL: viral load
