# Supplementary material for: An Unstructured Supplementary Service Data System for Daily Tracking of Patient Samples and Diagnostic Results in a Diagnostic Network in Malawi: System Development and Field Trial
Source: J Med Internet Res. 2021 Jul 6;23(7):e26582. doi: 10.2196/26582 (PMC8292942; doi:10.2196/26582)
Supplement: Multimedia Appendix 1 [file jmir_v23i7e26582_app1.docx]

## Multimedia Appendix 1

Summary statistics regarding the number of samples, by type, district, and facility, included in the USSD System Database from July 2019 to July 2020.

|  | **VL** | **EID** | **TB** | **Other** | **Total** |
| --- | --- | --- | --- | --- | --- |
| **Phalombe** | **19,354** | **1,405** | **569** | **27** | **21,355** |
| Nambazo Health Center | 2,317 | 67 | 62 | 5 | 2,451 |
| Kalinde Dispensary | 1,946 | 177 | 16 | 0 | 2,139 |
| Holy Family | 1,966 | 95 | 61 | 0 | 2,122 |
| Phalombe District Hospital | 1,730 | 163 | 0 | 0 | 1,893 |
| Sukasanje Health Center | 1,659 | 71 | 7 | 0 | 1,737 |
| Chitekesa Health Center | 1,529 | 146 | 21 | 0 | 1,696 |
| Migowi Health Center | 1,432 | 6 | 220 | 0 | 1,658 |
| Mkhwayi Health Center | 1,217 | 150 | 127 | 0 | 1,494 |
| Nkhulambe Health Center | 1,324 | 50 | 8 | 4 | 1,386 |
| Mpasa Health Center | 1,190 | 111 | 9 | 2 | 1,312 |
| Gogo Nazombe Health Center | 1,209 | 86 | 0 | 1 | 1,296 |
| Mwanga Health Center | 687 | 51 | 1 | 0 | 739 |
| Chiringa Maternity | 501 | 98 | 3 | 5 | 607 |
| Mulungu Alinafe | 394 | 106 | 0 | 8 | 508 |
| Chiringa Dispensary | 253 | 28 | 34 | 2 | 317 |
| **Salima** | **14,947** | **1,091** | **466** | **651** | **17,155** |
| Salima District Hospital | 3,720 | 294 | 0 | 0 | 4,014 |
| Khombedza Health Center | 1,553 | 81 | 43 | 0 | 1,677 |
| Chipoka Health Center | 1,465 | 59 | 24 | 18 | 1,566 |
| Lifeline Health Center | 1,407 | 48 | 58 | 14 | 1,527 |
| Thavite Health Center | 1,049 | 69 | 27 | 93 | 1,238 |
| Lifuwu Health Center | 1,073 | 122 | 10 | 11 | 1,216 |
| Maganga Health Center | 746 | 67 | 12 | 15 | 840 |
| Mchoka Health Center | 704 | 60 | 31 | 45 | 840 |
| Senga Bay Baptist Dispensary | 715 | 58 | 10 | 0 | 783 |
| Makiyoni Health Center | 458 | 23 | 64 | 124 | 669 |
| Ngodzi Health Center | 364 | 49 | 17 | 149 | 579 |
| Mafco Health Center | 579 | 19 | 28 | 7 | 633 |
| Chinguluwe Health Center | 336 | 31 | 24 | 28 | 419 |
| Katawa Health Center | 282 | 32 | 60 | 25 | 399 |
| Chitala Health Center | 190 | 20 | 29 | 0 | 239 |
| Parachute Health Centre | 92 | 5 | 0 | 93 | 190 |
| Chagunda Health Center | 113 | 18 | 21 | 18 | 170 |
| Kaphatenga Health Center | 101 | 36 | 8 | 11 | 156 |
| **Rumphi** | **6,657** | **483** | **1,824** | **1,378** | **10,342** |
| Rumphi District Hospital | 1,804 | 66 | 341 | 5 | 2,216 |
| Bolero Health Center | 1,847 | 125 | 155 | 29 | 2,156 |
| Lura Health Center | 506 | 19 | 83 | 319 | 927 |
| Mhuju Hospital | 533 | 52 | 51 | 49 | 685 |
| Katowo Rural Hospital | 518 | 43 | 41 | 73 | 675 |
| Jalawe Health Center | 3 | 4 | 257 | 244 | 508 |
| Chitsimuka Health Center | 0 | 0 | 465 | 3 | 468 |
| Nthenje Dispensary | 156 | 10 | 55 | 176 | 397 |
| DGM Livingstonia Hospital | 212 | 22 | 66 | 67 | 367 |
| Mwazisi Health Center | 244 | 29 | 57 | 8 | 338 |
| Chitimba Health Center | 226 | 26 | 0 | 62 | 314 |
| Ngonga Health Center | 128 | 15 | 78 | 68 | 289 |
| Mzokoto Health Center | 238 | 38 | 1 | 1 | 278 |
| Luwuchi Health Center | 121 | 14 | 3 | 92 | 230 |
| Mlowe Health Center | 41 | 5 | 67 | 45 | 158 |
| Mphopha Health Center | 35 | 6 | 62 | 42 | 145 |
| Tcharo Dispensary | 1 | 0 | 5 | 94 | 100 |
| Eva Demaya | 44 | 9 | 37 | 1 | 91 |
| **Grand Total** | **40,958** | **2,979** | **2,859** | **2,056** | **48,852** |
